# Effect of dose and dose rate on temporal γ-H2AX kinetics in mouse blood and spleen mononuclear cells in vivo following Cesium-137 administration

**DOI:** 10.1186/s12860-019-0195-2

**Published:** 2019-05-28

**Authors:** Helen C. Turner, Younghyun Lee, Waylon Weber, Dunstana Melo, Aimee Kowell, Shanaz A. Ghandhi, Sally A. Amundson, David J. Brenner, Igor Shuryak

**Affiliations:** 1 Center for Radiological Research, Columbia University Irving Medical Center, 630 West 168th Street, New York, NY 10032 USA; 2 Lovelace Biomedical, Albuquerque, NM 87108 USA; 3 Melohill Technology, Rockville, MD 20815 USA

**Keywords:** Radiation biodosimetry, Internal emitter, Radionuclide, Cesium-137, γ-H2AX

## Abstract

**Background:**

Cesium-137 (^137^Cs) is one of the major and most clinically relevant radionuclides of concern in a radiological dispersal device, “dirty bomb” scenario as well as in nuclear accidents and detonations. In this exposure scenario, a significant amount of soluble radionuclide(s) may be dispersed into the atmosphere as a component of fallout. The objectives of the present study were to investigate the effect of protracted ^137^Cs radionuclide exposures on DNA damage in mouse blood and spleen mononuclear cells (MNCs) in vivo using the γ-H2AX biomarker, and to develop a mathematical formalism for these processes.

**Results:**

C57BL/6 mice were injected with a range of ^137^CsCl activities (5.74, 6.66, 7.65 and 9.28 MBq) to achieve total-body committed doses of ~ 4 Gy at Days 3, 5, 7, and 14. Close to 50% of ^137^Cs was excreted by day 5, leading to a slower rate of decay for the remaining time of the study; ^137^Cs excretion kinetics were independent of activity level within the tested range, and the absorbed radiation dose was determined by injected activity and time after injection. Measurements of γ-H2AX fluorescence in blood and spleen MNCs at each time point were used to develop a new biodosimetric mathematical formalism to estimate injected activity based on γ-H2AX fluorescence and time after injection. The formalism performed reasonably well on blood data at 2–5 days after injection: Pearson and Spearman’s correlation coefficients between actual and predicted activity values were 0.857 (*p* = 0.00659) and 0.929 (*p* = 0.00223), respectively.

**Conclusions:**

Despite the complicated nature of the studied biological system and the time-dependent changes in radiation dose and dose rate due to radionuclide excretion and other processes, we have used the γ-H2AX repair kinetics to develop a mathematical formalism, which can relatively accurately predict injected ^137^Cs activity 2–5 days after initial exposure. To determine the assay’s usefulness to predict retrospective absorbed dose for medical triage, further studies are required to validate the sensitivity and accuracy of the γ-H2AX response after protracted exposures.

**Electronic supplementary material:**

The online version of this article (10.1186/s12860-019-0195-2) contains supplementary material, which is available to authorized users.

## Background

Cesium-137 (^137^Cs) is one of the major and most clinically relevant radionuclides of concern in a radiological dispersal device, “dirty bomb” scenario [1, 2] as well as in nuclear accidents [3] and detonations [4]. This exposure scenario may result in a significant amount of soluble radionuclides being dispersed into the atmosphere as a component of fallout, which could affect vast numbers of people located at substantial distances from the radiological event site. The Chernobyl and Fukushima Daiichi accidents as well as the Goiânia incident involving the theft of a forgotten ^137^CsCl radiotherapy source from an abandoned hospital site in the city of Goiânia in central Brazil, illustrate the large-scale response to environmental radioactive contamination [5–9]. Protracted low dose rate exposures, defined by UNSCEAR as being less than 0.1 mGy/min averaged over 1 h [10] can result from incorporation of radionuclides such as ^137^Cs into the body, e.g. by inhalation or ingestion of radioactive particles and/or contamination of wounds. The highly soluble chemical form, ^137^CsCl is rapidly absorbed from the gastrointestinal tract or lungs and permeates the entire body providing relatively uniform protracted beta particles and gamma irradiation [11, 12]. Cesium follows the same qualitative behavior as potassium after uptake. It is distributed throughout body tissues and is predominantly retained in the skeletal muscle tissue [13]. Irradiation of tissues can continue as long as the incorporated radionuclides remain in the body, which can last from weeks to decades depending on radionuclide type. Thus, in the event of a large-scale accidental or malicious release of volatile radionuclides, there is an important need to develop radiation biodosimetry assay(s) and technologies for population-based triage and subsequent dose-dependent medical management. At this time, it will be crucial to collect and analyze human biofluids (such as blood, urine, saliva) for accurate dose estimations and triage decision.

At the Columbia University Center for Medical Countermeasures against Radiation (CMCR) we have previously developed an internal-emitter mouse model for chronic whole-body irradiation using intraperitoneal, systemically distributed ^137^CsCl as a radiation source. Collectively, the teams investigated the long-term effects of ^137^CsCl activity for DNA damage [6] and global gene expresssion in peripheral blood cells [14] as well as metabolomic and lipidomic analyses to identify potential serum [15] and urinary [16] biomarkers of internal exposure, respectively. In these studies, C57BL/6 mice were injected with 8.0 ± 0.3 MBq of ^137^CsCl to produce cumulative total-body doses up to 10 Gy within 30 days after initial exposure which allowed us to evaluate total-body doses of interest for radiological triage (2–10 Gy) without inducing massive toxicity in the animals. Cesium activity is eliminated through excretion in the urine.

Cesium retention in the body is mathematically described by a sum of two exponential terms [17]. In mice, the first term of the equation represents mainly the elimination of about 20% of the administered ^137^Cs activity within about 0.6 day after its entry into blood. The second term of the equation reflects the remaining 80% of the administered ^137^Cs activity; which is retained in the tissues, more specifically skeletal muscle tissue. The second retention term is dominated by the skeletal muscle tissue because of the predominance of active transport in the cells of that tissue and the fact that muscle is a slowly exchanging tissue. It represents about 80% retained in the body with a biological half-time of 8 days for adult mice. Time-dependent changes in ^137^Cs activity in the body influences the dose rate, which is higher in the first day after ^137^Cs administration and decreases over time along with ^137^Cs excretion. The total-body dose also accumulates at a lower rate at the later time points which all add to the complexity of the prediction of the biological response.

Previously, we used the γ-H2AX biomarker to measure the effect of ionizing radiation on the induction and repair of DNA double strand breaks (DSBs) in blood lymphocytes in vivo after ^137^CsCl internal emitter exposure [6]. The γ-H2AX endpoint is an established radiosensitive biodosimetry biomarker, which has been used to assay DSBs in a variety of cell types and tissues after exposure to ionizing radiation [18–21]. H2AX, a member of the histone H2A family of proteins, is phosphorylated to form γ-H2AX foci at the site of radiation-induced DSBs [20, 22]. Immunofluorescent labeling of the γ-H2AX foci allows for direct visualization and quantitative measurement of DSBs in damaged cells. Measurement of the disappearance of radiation-induced γ-H2AX foci after radiation exposure provides an estimate of DNA repair over time. The key finding in our earlier study was that the γ-H2AX fluorescence signal in the blood lymphocytes remained elevated during the 30 days of ^137^Cs radionuclide incorporation, which is in clear contrast to acute exposures where most radiation-induced γ-H2AX foci typically resolve in 24–48 h [23–25]. Based on measurements of γ-H2AX yields at specific time points after initial ^137^Cs exposure, we developed a new mechanistically-motivated mathematical formalism to describe the interplay between increasing cumulative dose, decreasing dose rate during exposure, DSB repair, death of differentiated lymphocyte cells, and the production of new lymphocytes from damaged progenitor cells.

To validate our γ-H2AX model for protracted exposures, the objective of the present study was to examine in detail the effect of ^137^Cs activity, dose rate, and the accumulation of absorbed dose on γ-H2AX temporal kinetics in mouse blood and spleen mononuclear cells (MNCs) in vivo after ^137^Cs radionuclide incorporation. The study was designed using a range of ^137^CsCl injection activities (dose groups: 5.74, 6.66, 7.65 and 9.28 MBq) to target total-body committed doses of ~ 4 Gy on days 3, 5, 7, and 14 after exposure. The time frame was chosen as it is relevant to situations where radioactively-contaminated individuals would seek medical attention after a radiological event. The target total-body dose of ~ 4 Gy was chosen based on our earlier work, where on day 5 after administration of 8.0 ± 0.3 MBq ^137^CsCl: 1) γ-H2AX fluorescent levels were at their lowest, suggesting an increase in cell turnover and peak lymphocyte death [6]; 2) fewer differentially expressed genes were identified compared to the earlier and later time points, suggesting a general transition of transcriptional programs at or around this time [14] and, 3) an increased number of urine metabolites were more accurately separated compared to the control mice [16].

To apply biodosimetry to the important situation of protracted radionuclide exposures, we have extended our internal emitter in vivo mouse model by using four different ^137^Cs injection activities to develop a more complex model of doses/dose rates where some mouse groups received a similar dose rate but different doses while other groups received similar doses but different dose rates at triage-relevant time points, ranging from a few days to a few weeks of exposure. We present here a mathematical approach for biodosimetry of protracted internal radiation exposures based on 1) time (days), 2) committed absorbed total-body dose (Gy) and, 3) changes in dose rate (Gy/day) on γ-H2AX fluorescent yields in blood and spleen MNCs. We used this approach to estimate injected amounts of ^137^Cs based on γ-H2AX fluorescence in mouse blood and spleen mononuclear cells (MNCs), at specific times after injection.

## Methods

### Animals and irradiation

The animal studies were approved by the Lovelace Biomedical Institutional Animal Care and Use Committee (IACUC; approved protocol number FY15–087) and were conducted in facilities accredited by the Association for Assessment and Accreditation of Laboratory Animal Care (AAALAC). Male C57BL/6 mice (approximately 8–10 weeks old, 20–30 g) were purchased from Charles River Laboratories (Frederick, MD) and quarantined for a minimum of 14 days, 2 mice per cage in polycarbonate shoebox cages on racks with hardwood chip bedding. Animal pre-study body weight was used to randomize animals into five study groups consisting of 40 mice per group. Mice were dosed with a single, intraperitoneal (IP) injection of ^137^CsCl. Each group received different activities of ^137^CsCl; each group was then further divided into 5 subgroups based on length of time on study (Additional file 1: Table S1). Group 1 was a vehicle control group and did not receive ^137^CsCl, Group 2 received 5.74 MBq, Group 3 received 6.66 MBq, Group 4 received 7.65 MBq, and Group 5 received 9.28 MBq/animal. The same pH adjusted saline solution was used for the injection vehicle.

To accommodate the ^137^Cs administrations for 8 mice per data point across five dose groups (total 200 mice), three cohorts of C57BL/6 mice were prepared. Each cohort consisted of an equal number of animals from each dosing Study Group (based on administered activity) and each necropsy time point. Body weights of individual animals were ± 20% of the group means. After dose administration, ^137^Cs mice were single housed through to necropsy in disposable cages containing cardboard. Cages were separated by lead shielding to minimize cross-irradiation from adjacent mice. Control mice were left untreated and housed four mice per cage. All animals had unlimited access to Teklad Certified Global Rodent Diet 2016 (Harlan Teklad, Madison, WI) and water except during radionuclide administration and whole-body in vivo counting. Animals were examined twice per day (morning and afternoon) from arrival to the day of sacrifice. No abnormal observations were noted for any animal in any of the dose groups over the duration of the study.

### Whole body in vivo counting

Animals were placed in a small container (with numerous air holes) for approximately 5–10 min and whole body ^137^Cs content was measured using the Lovelace Biomedical’s in vivo photon counting system described previously in more detail in our companion studies [6, 14, 16]. We assumed a homogenous distribution for ^137^CsCl in the tissues. Mice were whole-body measured daily for the first 7 days and then again on days 10 and 14 post exposure. Vehicle controls were placed into similar containers for 5–10 min to ensure they were handled in the same manner as ^137^CsCl exposed animals. Measured whole body results were used to generate whole body retention curves for each animal. These curves were used to determine the radiation dose received by the mice. The calculation of radiation committed absorbed dose was calculated for each animal using the individual cesium parameters of the retention equation. Then the average committed absorbed dose based on the average whole body retention equation for each animal group was used in the analysis. The results of the animal measurements were transformed into activity using the calibration equation from the linear curve generated from phantoms of similar size and shape to the animals being measured. Linear curves were checked daily prior to the animal measurements.

### Mononuclear cell sample preparation

Mice were euthanized with an IP injection of Euthasol (1:9 dilutions with sterile saline). At necropsy, whole blood was collected by cardiac puncture into microtainer tubes with lithium heparin (#365971; BD Becton Dickinson & Co, Franklin Lakes, NJ). The dissected spleens were placed in 1.5 mL Eppendorf tubes containing 1 mL of phosphate buffered saline (PBS) with 2% fetal bovine serum (FBS) and kept at room temperature until cell isolation. Similar to our previous study protocols [6, 26], immediately after necropsy, whole blood samples, (~ 100–200 μl) were diluted with 5 volumes of RPMI-1640 medium (Invitrogen; Eugene, OR) and carefully layered over an equal volume of lymphocyte separation media (Histopaque-1083; Invitrogen). The samples were centrifuged at 1300 RPM for 30 min to form a lymphocyte band at the interface between the separation medium and plasma. The freshly isolated lymphocytes were washed twice with PBS and fixed with 2% paraformaldehyde (PFA) diluted in PBS and washed one more time. Each spleen was placed onto a 40-μm strainer (#352340; Fisher Scientific; Nazareth, PA) fitted to a 50 mL conical tube, pre-loaded with 10 mL 2% FBS/PBS at room temperature (RT). Using the tip of a syringe plunger (#309628; BD Becton Dickinson & Co) and 10 ml 1X PBS, the spleens were gently ground against the filter and cells filtered into the solution below. As with the blood samples, spleen MNCs were isolated using a ficol gradient (Histopaque-1083), washed and fixed with 2% PFA/PBS. All fixed samples were stored in PBS at 4 °C until the end of the study, after which all samples were shipped (4 °C) overnight by FedEx to the Center for Radiological Research, NYC.

### γ-H2AX immunocytochemistry

Each fixed-cell suspension was cytospun (Cytospin 4; Thermo Scientific, Waltham, Boston, MA) onto coated microscope slides (CYTOSLIDE™ #5991056; Thermo Scientific) and then placed into PBS. Immunodetection of γ-H2AX was performed according to our previously published protocols [6, 27], the cells were blocked with 3% bovine serum albumin (BSA; Sigma; St Louis, MO) for 30 min at room temperature and incubated with a rabbit polyclonal γ-H2AX (phospho S139) antibody (dilution 1:600; #ab2893 Abcam Inc., Cambridge, MA) for 2 h at RT, visualized with a goat anti-rabbit Alexa Fluor 488 (AF488) secondary antibody (dilution 1:1000; Invitrogen) and counterstained with DAPI in Vectashield® mounting medium (#H-1200; Vector Laboratories, Burlingame, CA). Images of cells were obtained using the Olympus epifluorescence microscope (Olympus BH2-RFCA). Fluorescent images of DAPI-labeled nuclei and AF488-labeled γ-H2AX were captured separately for each absorbed dose using the Olympus microscope and 60x oil immersion objective. Quantification of γ-H2AX yields was determined by measuring the total γ-H2AX nuclear fluorescence per lymphocyte and analyzed using image analysis software [21]. Similar to our previous work [6, 27], advanced apoptotic cells observed as a gross change in morphology of the DAPI-labeled nuclei, were not included for analysis by the image software program. For data acquisition, ~ 150–500 paired lymphocyte images were captured and analyzed per data point.

### Mathematical formalism

We developed a simple mathematical formalism to describe the effects of injected radioactivity (*A*) and time (*t*) on γ-H2AX fluorescence data (*F*) using plausible hypotheses about the underlying physical and biological processes. This formalism is new in design, but shares some concepts with our earlier work [6]. It is represented by the following equation:

1 $$ F=b+k\times A\times t\times \exp \left[{Q}_1+{Q}_2\right],{Q}_1=-\alpha \times A,\kern0.5em {Q}_2=1-{\left(1+r\times t\right)}^p $$

Here parameter *b* represents background γ-H2AX fluorescence in unirradiated controls. The term *k* × *A* × *t*, which contains the parameter *k*, represents an initial radioactivity-dependent increase in γ-H2AX levels when irradiation starts and the dose rate is high. The term *Q*_1_, which contains the parameter α, represents a decrease in γ-H2AX levels due to the death of heavily radiation-damaged cells. The term *Q*_2_, which contains the parameters *r* and *p*, represents time-dependent decrease in γ-H2AX levels due to radionuclide excretion, DNA repair, and death of heavily damaged cells. The mathematical structure of term *Q*_2_ is based on a modified stretched exponential function, which is useful for describing complex decay patterns using small numbers of parameters [28].

### Fitting procedure and estimation of parameter uncertainties

Our formalism (Eq. 1) contains 5 parameters (*b*, *k*, α, *r*, *p*). The background parameter *b* was fixed at the measured value of control level arbitrary fluorescence units. Best-fit values for the remaining 4 parameters were obtained by fitting Eq. 1 to the data by maximum likelihood estimation using nonlinear weighted regression, assuming Gaussian errors and weights based on standard errors of the data points. Fitting was performed by the sequential quadratic programming (SQP) algorithm [29] in Maple 2017® software. Uncertainties (95% confidence intervals, CIs) for each adjustable parameter were estimated by profile likelihood.

### Procedure for predicting injected radioactivity based on γ-H2AX fluorescence and time

Measurements of γ-H2AX fluorescence have important potential as biodosimetry tools, where the goal is to estimate the magnitude of a subject’s radiation exposure (e.g. the amount of radioactivity incorporated into the body and/or the resulting cumulative radiation dose) based on the time after a radiological incident and the γ-H2AX fluorescence. To assess the potential utility of the data set and formalism presented here for biodosimetry purposes, we performed a Monte Carlo simulation-based procedure using Maple 2017® software. This procedure used the best fit (Eq. 1) as a “calibration curve” to estimate radioactivity level, and relied on Monte Carlo simulations to explore the uncertainties of these estimates. It consisted of the following steps:

1. Multiple (> 5000) parameter combinations (i.e. values of *k*, α, *r*, *p*) that resulted in fits to the data that were within the 95% confidence region of the best fit were identified by randomly exploring the parameter space in the vicinity of the best-fit parameter combinations. We denote each such parameter combination generated by the *i*-th Monte Carlo simulation by *ψ*_*i*_.
2. Each parameter combination (*ψ*_*i*_), along with the value of the background parameter *b* and the time *t*_*j*_ for the *j*-th data point (e.g. 2 days after radioactivity administration), were substituted into the formalism (Eq. 1).
3. These substitutions produced the function *F*(*ψ*_*i*_, *t*_*j*_, *A*_*i*_*,*_*j*_), which predicts the γ-H2AX fluorescence (*F*) based on the parameter combination (*ψ*_*i*_), the time (*t*_*j*_), and the injected radioactivity (*A*_*i*_*,*_*j*_).
4. For biodosimetry purposes, our goal was to find the value of *A*_*i*_*,*_*j*_ in *F*(*ψ*_*i*_, *t*_*j*_, *A*_*i*_*,*_*j*_). This was done by minimizing the following function *f*_*i*_*,*_*j*_, where *F*_*j*_ is the measured γ-H2AX fluorescence for the *j*-th data point:

2 $$ {f}_{i,j}={\left[F\left({\psi}_i,{t}_j,{A}_{i,j}\right)-{F}_j\right]}^2 $$

Implementing steps 1–4 resulted in an estimated radioactivity value (*A*_*i*_*,*_*j*_) based on the following inputs: time (*t*_*j*_), measured γ-H2AX fluorescence (*F*_*j*_), and parameter combination (*ψ*_*i*_). Median, minimum and maximum values of *A*_*i*_*,*_*j*_ across all Monte Carlo simulations (i.e. across all values of *i*) were compared with measured radioactivity values at each (*j*-th) data point (*A*_*j*_) to assess how close the calculated estimates were to the real data.

In addition to these analyses of injected activity as a continuous variable, we performed a binary classification analysis where the injected activity was categorized as “low” (for the two lowest values, 5.74 and 6.66 MBq) or “high” (for the two highest values, 7.65 and 9.28 MBq). The injected activity predicted by the model was converted to a 0–1 scale by dividing each value by the maximum over all mouse groups. From these data, a receiver operating characteristic (ROC) curve was constructed in *R* 3.5.2 software, using the 2019 *pROC* package (https://cran.r-project.org/web/packages/pROC/pROC.pdf), to assess the model’s performance in classifying injected activity into “low” vs “high” categories.

### ^137^Cs dose and dose-rate calculation

The cesium retention in the whole-body is mathematically represented by a sum of two exponential terms. The parameters of the whole-body retention equation were calculated for each one of those 160 animals in the study. About 20% of the administered activity is retained with a biological half-time of 0.6 d and 80% is retained with a biological half-time of 7.8 d (Eq. 3). For the animals allocated in the groups with short duration studies, just one exponential term is observed. The parameters of retention equation for each animal were used to calculate the total number of disintegration of ^137^Cs in the animal’s body. In order to calculate the dose-rate for each day post ^137^Cs administration, the total number of disintegration was calculated for each time-period post ^137^Cs injection (1d, 2d, 3d, …. 14d).

3 $$ \mathrm{R}\left(\mathrm{t}\right)=0.20{\mathrm{e}}^{-\left(\frac{0.693}{0.6}\right)\mathrm{t}}+0.80{\mathrm{e}}^{-\left(\frac{0.693}{7.8}\right)\mathrm{t}} $$

The dose coefficient (Gy.Bq^− 1^ of administered activity) was calculated for each day following ^137^Cs administration, multiplying the total number of disintegration by the specific effective energy (SEE) value for ^137^Cs (2.41 × 10^− 19^Gy.dis^− 1^).

The dose coefficient is calculated using the Eq. (4)

4 $$ \frac{D}{A}={\int}_{t_0}^{t_{0+t}}\overset{\check{} }{A}(s)\times SEE\left(T\leftarrow S\right)\kern0.5em \left(\frac{Gy}{Bq}\right) $$

Where: $\overset{\sim }{A}(S)$ is the total number of disintegrations that will occur within source organs/tissues overtime, SEE (T ← S) is the specific effective energy in MeV kg^− 1^, i.e., it is the energy imparted per kilogram of a target tissue (T) as a consequence of a disintegration of ^137^Cs in source organs/tissues (S) = 2.41 × 10^−19^Gy/dis. The committed doses for each day post ^137^Cs administration were calculated by multiplying the administered activity by the dose coefficient for that given day (Eq. 5). The dose-rate for a given day was calculated by subtracting the committed dose calculated for that given day from the one from the previous day.

5 $$ D=\frac{D}{A}\times A\kern0.5em (Gy) $$

where, D = Absorbed dose (Gy); D/A = dose coefficient; A = administered activity (Bq).

## Results

### Biokinetics and dosimetry

**Fig. 1:**
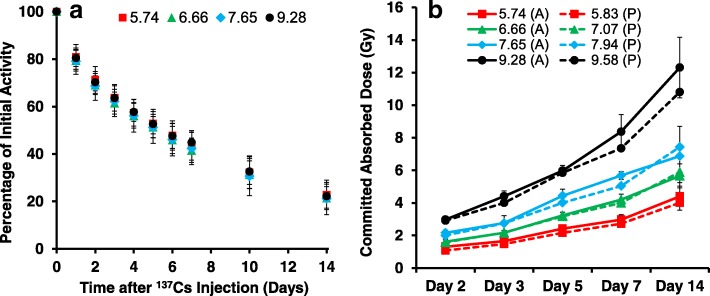
Panel **a** Whole-body counting data normalized to the amount of ^137^Cs present in each animal on Day 0 following injection. For each dose group (in MBq: 5.74, 6.66, 7.65 and 9.28), the percentage of original injected activity remaining is plotted as the mean average (*n* = 8 mice) at each time point. Panel **b** the actual (A) and predicted (P) total body absorbed dose for each day of sacrifice. Error bars denote standard deviation

All animals remained in apparent good health, with no adverse events observed during the course of the study. Figure 1a shows the average ^137^Cs retention curve (*n* = 8 mice per data point) for each of the four injection dose groups used to calculate the whole-body committed dose to each dose group of animals during the 14 day study period. Presented as a percentage of the injected activity remaining, the rates of radionuclide decay were similar and close to exponential, indicating that excretion rates are independent of administered activity. The results show that close to 50% of the radionuclide was excreted by day 5, leading to a slower rate of decay for the remaining time of the study. Using individual whole body retention curves, the dose to each mouse was calculated. Figure 1b shows the actual (A) measured and predicted (P) average (n = 8 mice) total body absorbed doses (Gy) for each dose group, calculated at specific time points following ^137^Cs injection. The results show that whole-body radiation doses cumulated up to 12.3 Gy during the 14-day study period. The calculated average injected dose activity was 103% of the target and, dose group averages were within 20% of the targeted dose, indicating that the retention profiles for the current study closely matched the retention profiles in our previously conducted study [6, 14, 16]. Table 1 summarizes the average absorbed dose and dose rates (Gy/day) measured over the 14 day study period.

**Table 1:** Accrued total-body dose (Gy) and dose rates (Gy/day) measured over the 14-day study period

Administered Activity (MBq)	Whole-body Committed Dose (Gy)				
	2 days	3 days	5 days	7 days	14 days
5.74	1.31 ± 0.36	1.65 ± 0.07	2.42 ± 0.14	2.97 ± 0.31	4.3 ± 0.84
6.66	1.62 ± 0.57	2.16 ± 0.57	3.23 ± 0.20	4.2 ± 0.34	5.61 ± 0.70
7.65	2.16 ± 0.17	2.76 ± 0.45	4.44 ± 0.41	5.68 ± 0.24	6.81 ± 1.84
9.28	2.98 ± 0.14	4.41 ± 0.32	5.98 ± 0.32	8.37 ± 1.05	12.31 ± 1.86
Administered Activity (MBq)	Dose Rates (Gy/day)				
	2 days	3 days	5 days	7 days	14 days
5.74	0.61 ± 0.17	0.47 ± 0.02	0.39 ± 0.03	0.3 ± 0.04	0.16 ± 0.05
6.66	0.74 ± 0.07	0.6 ± 0.17	0.52 ± 0.03	0.42 ± 0.04	0.201 ± 0.04
7.65	0.97 ± 0.08	0.78 ± 0.11	0.69 ± 0.07	0.58 ± 0.03	0.24 ± 0.11
9.28	1.36 ± 0.07	1.25 ± 0.08	0.94 ± 0.05	0.85 ± 0.08	0.44 ± 10

### γ-H2AX kinetic profile in peripheral blood mononuclear cells (PBMCs)

**Fig. 2:**
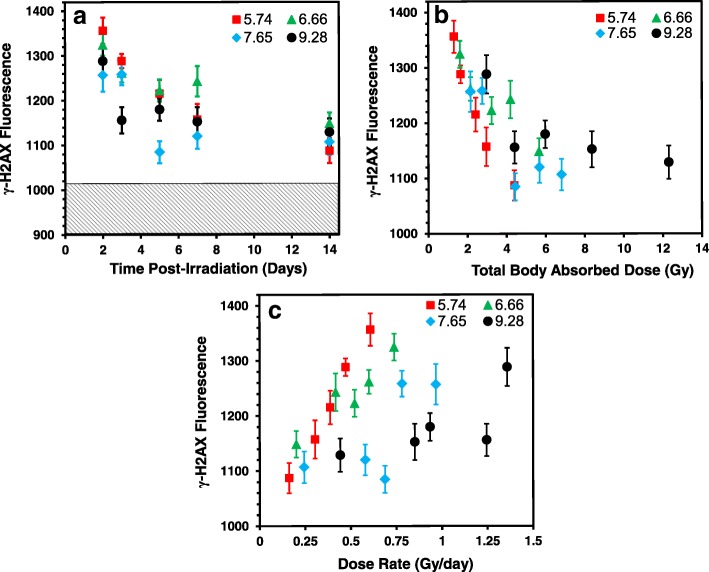
Mean response pattern of γ-H2AX yields after 2–14-day internal exposures to ^137^CsCl (injection group activity in MBq: 5.74, 6.66, 7.65 and 9.28). As a function of **a** time (days), **b** accrued total-body dose (Gy) and **c** dose rate (Gy/day)

Figure 2 shows the mean γ-H2AX nuclear fluorescence values measured in peripheral blood MNCs relative to the unirradiated control cells as function of time (Fig. 2a), accrued total-body dose (Fig. 2b) and dose rate (Fig. 2c) following ^137^CsCl injection (in MBq: 5.74, 6.66, 7.65 and 9.28). Median values (not shown) showed a similar temporal pattern, such that mean: median γ-H2AX yields were ~ 1:1. The fluorescence data, as the average pixel value for every scored nucleus was edited for high intensity γ-H2AX fluorescence labeling (total fluorescence > 3000 arbitrary fluorescence units) due to fragmented nuclear DNA [30]. Mean (± SEM) γ-H2AX fluorescence data for the ^137^Cs groups and sham-treated controls over the entire study period can be found in Additional file 2: Table S2. The results show that throughout the 14-day study period, γ-H2AX levels in blood MNCs from the ^137^Cs injected mice remained above non-irradiated, background levels. The mean γ-H2AX levels in the sham-treated mice showed a maximum of 1026 ± 35 and a minimum of 972 ± 42 with 5.3% variation between each group. Given that the γ-H2AX control, background levels were relatively constant over time, Fig. 2a shows overall mean (1006 ± 36) level for the control mice over the study period (hatched area).

**Fig. 3:**
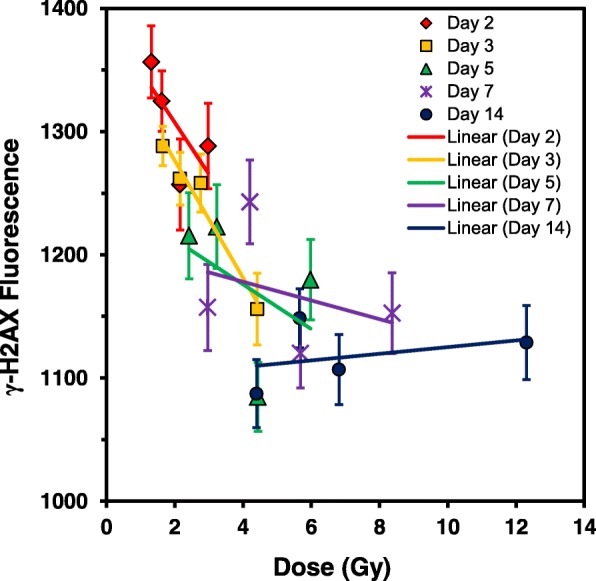
Linear regression analysis of the γ-H2AX dose-response. Data points represent mean γ-H2AX fluorescent yields for each ^137^CsCl activity group measured at each time point where each day is colour-coded

The data show that for all four ^137^Cs injected activities, there was an overall decrease in γ-H2AX yields with increasing time (Fig. 2a) and accrued total body dose (Fig. 2b). Peak γ-H2AX levels were apparent in blood samples collected on day 2 with committed absorbed doses ranging 1.3–2.8 Gy across the four radionuclide activities. For the lowest injected activity used here, 5.74 MBq, γ-H2AX yields declined with increasing time with the committed absorbed dose reaching a nadir at 4.4 Gy (day 14), and similarly for 6.66 MBq, γ-H2AX levels continued to decrease up to 5.7 Gy (day 14). For the two highest ^137^Cs activities, 7.65 and 9.28 MBq, γ-H2AX yields rapidly decreased after an accrued dose of 4.4 Gy on days 5 and 3, respectively. By day 14, γ-H2AX yields showed significant overlap between the different ^137^CsCl-injected activities. As radionuclide dose-rate decreases over time due to increased ^137^Cs excretion, the dose response of γ-H2AX is harder to detect. When total γ-H2AX fluorescence levels in the blood MNCs were plotted against dose rate (Fig. 2c), a dose-rate effect was only observed for the two lowest injection activities 5.74 and 6.66 MBq, observed as a monotonic increase in γ-H2AX yields with increasing dose rate; whereas for the higher injection activities, 7.65 and 9.28 MBq, the γ-H2AX slopes were flatter. Linear regression analysis (Fig. 3) shows that the γ-H2AX response decreases with increasing dose and time. At the earlier time points (days 2 and 3), γ-H2AX levels decreased rapidly with increasing dose, whereas at the later time points, the dose response is reduced and flatter. By day 14, γ-H2AX levels may increase with increasing dose, evidence for perhaps an increase in DNA damage in newly formed cells/repopulated cells.

### Mathematical formalism to predict the amount of ^137^Cs activity

**Fig. 4:**
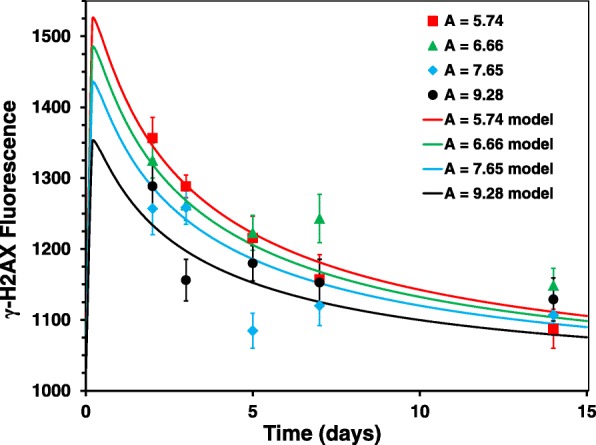
Comparison of best-fit curves (lines) with measured γ-H2AX fluorescence (symbols). Error bars represent standard errors. Each data point represents the mean γ-H2AX fluorescence from each 8-mouse dataset after injection with 5.74, 6.66, 7.65 and 9.28 MBq of ^137^CsCl at day zero. This detailed activity information was used in fitting our mathematical formalism, but the plotted curves represent best-fit predictions for the average activity at day zero (A, in MBq) across 4 mouse groups labeled with the same color as the curve

Despite the complicated nature of the studied biological system and the time-dependent changes in radiation dose and dose rate due to radionuclide excretion and other processes, our simple mathematical formalism (see Eq. [1] in Methods) provided a reasonable fit to the data sets (Fig. 4). Specifically, the formalism reproduced the following patterns that were seen in the current data and/or expected based on prior knowledge of radiation-induced γ-H2AX kinetics: (1) γ-H2AX fluorescence increased markedly above background values shortly after radiation exposure began. The magnitude of this increase depended on injected radioactivity. (2) γ-H2AX fluorescence decreased rapidly over the first week of exposure, but the rate of decrease slowed down with time. Best-fit parameters and their uncertainties are shown in Table 2.

**Table 2:** Best-fit parameter values for our mathematical formalism

Parameter	Affected process	Best-fit value	95% CIs	
k (MBq^− 1^ × days^− 1^)	Initial increase in γ-H2AX levels when irradiation starts	4.65 × 10^5^	3.28 × 10^5^	6.60 × 10^5^
α (MBq^−1^)	Decrease in γ-H2AX levels due to death of heavily damaged cells	0.255	0.183	0.323
r (days^−1^)	Time-dependent decrease in γ-H2AX levels due to radionuclide excretion, DNA repair, and death of heavily damaged cells	1.07 × 10^6^	7.54 × 10^5^	1.52 × 10^6^
p (unitless)		0.153	0.146	0.159

**Fig. 5:**
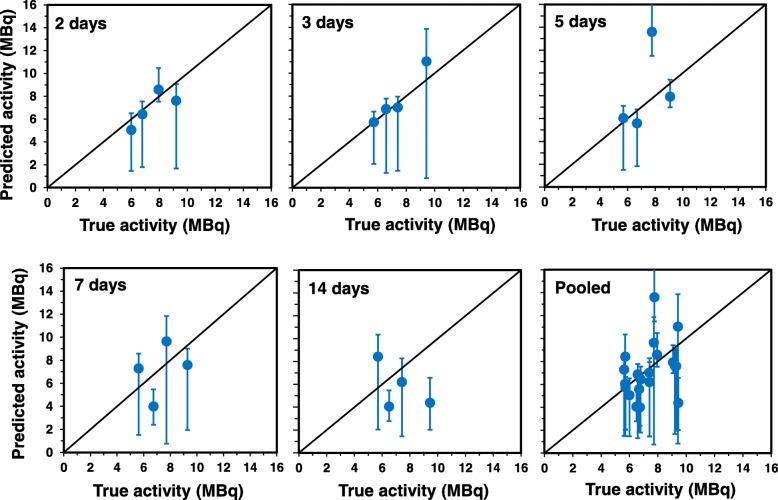
Comparisons of true injected values ^137^Cs activities (x-axis) and calculated estimates (symbols) at different time points (y-axis). Solid circles represent median calculated radioactivity estimates across multiple Monte Carlo simulations, and error bars indicate their ranges (minimum to maximum). The black line represents a theoretical 1:1 correspondence. Details are described in the main text

Comparisons of true injected radioactivity values and calculated estimates at different time points are shown in Fig. 5. Because injected activity determines absorbed dose, both the true and estimated activity values were converted to doses using time-dependent conversion coefficients based on Table 1. These results are shown in Additional file 3: Figure S1, and of course show the same patterns as Fig. 5, but with dose rather than activity as the chosen metric. Good correlations between true values and estimates were obtained during the first 5 days of exposure (Table 3). At longer times, the predictive ability of the formalism decreased, probably because the separation between γ-H2AX fluorescence values by radioactivity levels visibly decreased with time (Fig. 4). Specifically, at the longest measured time point of 14 days the γ-H2AX values from all 4 radioactivity levels were essentially overlapping.

**Table 3:** Correlations between true injected radioactivity values and median estimates calculated using our mathematical formalism over different time ranges. Details are described in the main text

Time	Pearson correlation coefficient	*p*-value	Spearman’s correlation coefficient	*p*-value
2–3 days	0.857	0.00659	0.929	0.00223
2–5 days	0.610	0.0350	0.804	0.00161
2–7 days	0.539	0.0312	0.691	0.00302
2–14 days	0.337	0.147	0.380	0.0980

Receiver operating characteristic (ROC) analysis was used for the evaluation of the model’s performance in discriminating between “high” and “low” injected activities (Additional file 4: Figure S2). The area under the ROC curve was 0.93, with 95% CI of 0.806–1.0 (by the DeLong method [31]). Based on this result, the model performed well in classifying samples into the “low” vs “high” injected activity categories based on γ-H2AX values.

### γ-H2AX levels in spleen mononuclear cells (MNCs)

Using the same formalism that we developed above for blood, analysis of the γ-H2AX yields in the spleen mononuclear cells did not show good prediction of the injected activity. Only the 14 day time point was meaningfully fitted by the formalism such that a monotonic relationship was observed between γ-H2AX and injected activity. The correlation coefficients between real and estimated activities at day 14 were: Pearson correlation coefficient 0.866, *p* = 0.134, Spearman’s correlation coefficient 1.0, *p* = 0.083. These correlations are good, but did not achieve statistical significance because there are only 4 activity values to compare.

**Fig. 6:**
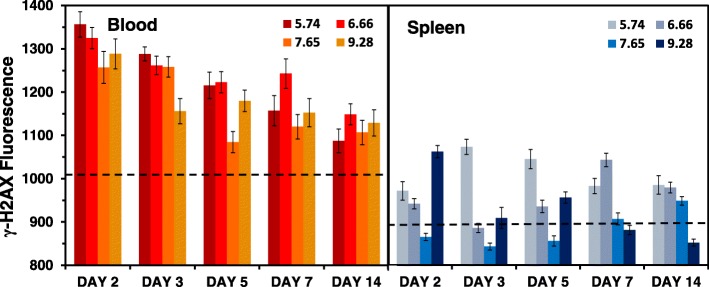
Mean γ-H2AX nuclear fluorescence levels in blood and spleen MNCs. Data show means ± SEM. Dashed lines show the mean γ-H2AX levels in non-irradiated mice across the 14-day study period

Figure 6 compares the mean total γ-H2AX nuclear fluorescence levels in blood and spleen MNCs for dose and time after ^137^CsCl administration. The dashed lines show the non-irradiated, background levels in blood (1006 ± 36) and spleen (878 ± 18) MNCs. The results indicate that in general there are higher levels of γ-H2AX DSBs in blood MNCs compared to the spleen MNCs across the 14-day study period. For each sample, we also evaluated the number of MNCs with high levels of γ-H2AX (cut-off > 3000 arbitrary units; interpreted as “highly damaged/dying” cells) or low levels of γ-H2AX (cut-off < 500 arbitrary units; interpreted as “healthy or newly formed” cells). For ease of interpretation we have presented the ratios both ways (Additional file 5: Table S3). With no obvious time course, the data indicate that the spleen MNCs appear less damaged than the blood. The results also show an apparent larger number of dying/apoptotic cells in circulating blood and spleen MNCs on days 2 through 7 after injection with the two lowest activities as opposed to the two highest activities, where the heavily damaged cells may have already died and been removed from the circulation, leading to an apparent increased number of healthy/damaged cells.

## Discussion

It is likely that ^137^Cs is the most biologically important fission product from a nuclear accident scenario [1] and thus, following a large-scale accidental or malicious release of ^137^Cs there is a need to develop radiation biodosimetry assays and technologies for population-based triage and medical management. In our earlier work, we used the γ-H2AX biodosimetry marker to show qualitative differences in the response to acute and protracted ^137^Cs irradiation. The fact that the γ-H2AX signal remains persistently elevated above non-irradiated, control levels during radionuclide exposure allowed us to mathematically describe the temporal γ-H2AX response. The challenge here was to apply this important situation of complex protracted irradiation and dose/dose rate effects to biodosimetry and validate the γ-H2AX biomarker to estimate radionuclide exposure activity, which determines absorbed dose.

To assess total-body full dose/dose rate implications, the present study was designed using four injection ^137^Cs activities of 5.74, 6.66, 7.65 and 9.28 in MBq such that some mouse groups received a similar dose rate but different doses while other groups received similar doses but different dose rates at triage-relevant time points, ranging from a few days to a few weeks of exposure (Additional file 1: Table S1). Mouse whole body retention curves showed that the ^137^CsCl decay kinetics across the range of ^137^CsCl activities were similar, indicating that the excretion rates are independent of injected activity (Fig. 1a). The same was observed among the people contaminated with ^137^Cs in the Goiania accident [13]. Whole-body radiation doses up to 12.3 Gy were delivered after 14 days of radionuclide exposure (Fig. 1b), confirming that total-body absorbed dose is dependent on injection activity and time. Measurements of γ-H2AX fluorescent yields in blood MNCs appear highly dependent on time (Fig. 2a) and dose (Fig. 2b) during the 14 day study period. The γ-H2AX DNA damage response decreases with increasing time and committed total-body absorbed dose such that dose is a surrogate for time. As the dose-rate decreased due to increased ^137^Cs excretion, the dose response for γ-H2AX protein yields is harder to detect. A dose-rate effect (Fig. 2c) was observed for only the two lowest injection activities 5.74 and 6.66 MBq, observed as a monotonic increase in γ-H2AX yields with increasing dose rate.

We have developed a mechanistically-motivated mathematical formalism of γ-H2AX kinetics and fitted it to the γ-H2AX response during protracted exposures (Fig. 4). The formalism assumes that γ-H2AX fluorescence increases rapidly in a dose-dependent manner after internal emitter exposure begins, and then slowly decreases in a stretched-exponential manner. It seeks to describe the net effects of DNA damage response activation, cell turnover, and elimination of radioactive cesium from the body by excretion and physical decay. The best fits suggest that peak γ-H2AX levels may have occurred prior to Day 2, at which time the circulating mature T-cells are exposed to the largest dose rates following the administration of a single dose of the ^137^Cs radionuclide. The decay pattern shows that after their early peak, the γ-H2AX levels continue to decrease with increasing time, which is likely due to a combination of increased apoptotic cell death of the damaged blood lymphocytes, DNA repair and the slower formation of new DNA damage. Furthermore, the γ-H2AX response measured at targeted whole-body doses of ~ 4 Gy, achieved at different time points showed no apparent correlation with injected activity, suggests that the time of radionuclide exposure is an apparent dominant factor in the peak death of highly damaged cells.

Monte Carlo simulation procedures estimated injected radioactivity, which ultimately determines absorbed dose, using time and measured γ-H2AX fluorescence as inputs (Table 2). The results show that dose predictions using the formalism are better at the beginning, particularly over 2–3 days whereas after 5 days, prediction accuracy falls off (Table 3). The Monte Carlo simulations also indicated that the two lowest activity datasets were able to better predict the injected dose compared to the two highest activities. These results suggest that the most robust effects of ^137^Cs-induced γ-H2AX formation may occur relatively soon after exposure because at this time the dose rates are maximal and many of the heavily damaged cells have not yet died. At the highest ^137^Cs radioactivity levels some saturation of the γ-H2AX response may occur. At longer times, when dose rates decrease and heavily damaged cells are eliminated and replaced by newly-formed cells, the differences in response to different radioactivity levels become less marked and more difficult to detect. Nevertheless, ROC analysis based on data at all time points (Additional file 4: Figure S2) showed good discrimination between “high” and “low” injected activities.

Cell death/apoptosis measurements are important physiological biomarkers of the radiation-induced injury response. The fact that an inverse response is produced at 2 days, might suggest that the approach for γ-H2AX analysis may be open to misinterpretation. To assess the usefulness of the γ-H2AX approach and improve the accuracy of ^137^Cs internal exposure activity for medical triage, future studies should aim to: 1) expand and validate the γ-H2AX response for lower dose/rate chronic radionuclide exposures (e.g. from 0 to 2 Gy) using both γ-H2AX foci number vs. fluorescent intensity analysis methods, and 2) develop a panel of lymphocyte biomarkers for use in MNC lymphocyte subsets. Studies by Horn and colleagues [32] have shown that the combined analysis of γ-H2AX/53BP1 foci and caspase activation in lymphocyte subsets may provide a rapid and more accurate triage tool in recent and more remote radiation exposures. In the present work, highly fluorescent MNCs that are qualitatively determined as being apoptotic were removed from each data set. For example, for the lowest ^137^Cs injection activity, the results indicated an apparent increase in cells undergoing apoptosis up to 5 days (absorbed dose = 2.42 Gy) that decreased by 7–14 days. The inclusion of these “dying” cells to the overall mean γ-H2AX measurements could potentially influence and change the shape of DNA damage γ-H2AX kinetics curve. In future studies, we plan to use our low dose rate gamma irradiator (**VA**riable **D**ose- rate **E**xternal i**r**radiato**R**, VADER [1]) to investigate these observations further.

Considering the relatively small sample size and small volumes (100–200 μl) of blood used here, we expanded the study to investigate γ-H2AX yields in matched spleen MNCs. Unlike the blood MNC datasets, the results showed an undeterminable correlation for γ-H2AX yields with dose and dose rate, which understandably lead to poor dose predictions. Differences between the two tissues were further highlighted when we directly compared the mean γ-H2AX levels across each data point (Fig. 6) and assessed the numbers of apparent dying/apoptotic cells to healthy cells during ^137^Cs exposure (Additional file 5: Table S3). The finding that the spleen appears to host a reservoir of apparently healthier MNCs compared to the blood, implies that the cell environment might contribute to the DNA damage response in blood and spleen MNCs following protracted, low dose-rate ionizing irradiation across the 14-day study period. Studies by Pecaut et al. [33], that investigated the dose and dose rate effects of whole-body gamma-irradiation in lymphocytes and lymphoid organs, suggested that cells in the mouse spleen are more effected by dose rate than those in the blood. They concluded that the response of lymphocytes in different body compartments may be variable.

There is a need for simple, high throughput and reliable cytogenetics assays which in the event of a mass-casualty radiological incident, could also be used in combination with standard internal dosimetry methods based on excreted systemic activity and biokinetic modelling. The advantage of combining these methods for medical triage is that γ-H2AX and cell death biomarkers also reflect biological damage and injury in blood as opposed to just using purpose-built mobile portal monitors to measure the physical activity of gamma emitters [34]. As far as we are aware, few studies to date have developed models for protracted, continuous, internal whole-body irradiation model to evaluate absorbed dose and quantify DNA damage after radionuclide administration [35, 36]. Recent studies by Scherthan and Lassmann [36, 37] measured the induction, persistence, and disappearance of radiation-induced γ-H2AX and 53BP1 foci after the first ^131^I therapy of patients with differentiated thyroid carcinoma. Their results suggest that the γ-H2AX/53BP1 could be useful biomarkers for detecting low dose radiation exposure and measurement of DNA damage repair kinetics after radionuclide incorporation.

## Conclusions

We have used the γ-H2AX repair kinetics to develop a mathematical formalism, which can relatively accurately predict injected ^137^Cs activity 2–5 days after initial exposure. The advantage of the γ-H2AX biodosimetry assay is that it is highly adaptable to high throughput technologies [38–40] using small volumes of blood [21, 41] which offers a rapid and minimally invasive approach to estimate the amount of incorporated radionuclides in subjects exposed to accidental or malicious radiological events, days after initial exposure. The results presented here warrant further studies to validate the accuracy and reliability of the γ-H2AX biomarker response in a protracted, internal exposure scenario. Future studies at the Columbia Center for High Throughput Minimally Invasive Radiation Biodosimetry Center, will use our newly developed low dose rate gamma irradiator VADER, which can provide arbitrarily varying and constant low dose rate irradiations in the range of 0.05 to 1 Gy/day [1]. The advantages of such a system are that it will allow for simpler experiments in a controlled, non-radioactive setting to decouple the time dependence of delivered dose/dose rate from the biokinetics in the animal model used. Furthermore, protocols for mouse model irradiations and biodosimetry measurements could also be developed for a temporal dose profile likely to be experienced by human real life scenarios including mixed injury [1, 13, 42, 43].

## Additional files

Additional file 1: **Table S1.** Mouse Experimental Design. (PDF 11 kb)

Additional file 2: **Table S2.** Mean γ-H2AX total fluorescence yields in blood and spleen MNCs. (PDF 72 kb)

Additional file 3: **Figure S1.** Dose predictions calculated from injected ^137^Cs activity predictions at different time points. Solid circles represent median estimates across multiple Monte Carlo simulations, and error bars indicate their ranges (minimum to maximum). The black line represents a theoretical 1:1 correspondence. (PDF 140 kb)

Additional file 4: **Figure S2.** ROC analysis of model performance on “low” vs “high” injected activities (AUC = 0.93). (PDF 4 kb)

Additional file 5: **Table S3.** Measurement of the number of healthy and highly- damaged blood and spleen MNCs over the 14-day study period. For comparison, the ratio of damaged/healthy cells in non-exposed blood and spleen MNCs were 0.1; whereas the ratios of healthy/damaged cells were 17.6 and 12.3, respectively. (PDF 5 kb)

## Abbreviations

DSBs: Double strand breaks
MNCs: Mononuclear cells
SQP: Sequential quadratic programming
VADER: VAriable Dose-rate External irradiatoR
