# Supplementary material for: Effect of dose and dose rate on temporal γ-H2AX kinetics in mouse blood and spleen mononuclear cells in vivo following Cesium-137 administration
Source: BMC Mol Cell Biol. 2019 May 28;20:13. doi: 10.1186/s12860-019-0195-2 (PMC6540459; doi:10.1186/s12860-019-0195-2)
Supplement: Supplementary file 1 — Table S1. Mouse Experimental Design. (PDF 11 kb) [file 12860_2019_195_MOESM1_ESM.pdf]

Study Group (Animal IDs)	Study Subgroup (Animal IDs)	N	$^{137}CsCl$ Activity (Targeted)	In vivo Counts (days)	Necropsy (days)
Group 1 (1001-1040)	1a (1001-1008)	8	n/a[1]	0-2	2
	1b (1009-1016)	8		0-3	3
	1c (1017-1024)	8		0-5	5
	1d (1025-1032)	8		0-7	7
	1e (1033-1040)	8		0-7, 10, 14	14
Group 2 (2001-2040)	2a (2001-2008)	8	5.74 MBq	0-2	2
	2b (2009-2016)	8		0-3	3
	2c (2017-2024)	8		0-5	5
	2d (2025-2032)	8		0-7	7
	2e (2033-2040)	8		0-7, 10, 14	14
Group 3 (3001-3040)	3a (3001-3008)	8	6.66 MBq	0-2	2
	3b (3009-3016)	8		0-3	3
	3c (2017-3024)	8		0-5	5
	3d (3025-3032)	8		0-7	7
	3e (3033-3040)	8		0-7, 10, 14	14
Group 4 (4001-4040)	4a (4001-4008)	8	7.65 MBq	0-2	2
	4b (4009-4016)	8		0-3	3
	4c (4017-4024)	8		0-5	5
	4d (4025-4032)	8		0-7	7
	4e (4033-4040)	8		0-7, 10, 14	14
Group 5 (5001-5040)	5a (5001-5008)	8	9.28 MBq	0-2	2
	5b (5009-5016)	8		0-3	3
	5c (5017-5024)	8		0-5	5
	5d (5025-5032)	8		0-7	7
	5e (5033-5040)	8		0-7, 10, 14	14

[1]Animal received a pH matched saline injection
