# Supplementary material for: Effect of dose and dose rate on temporal γ-H2AX kinetics in mouse blood and spleen mononuclear cells in vivo following Cesium-137 administration
Source: BMC Mol Cell Biol. 2019 May 28;20:13. doi: 10.1186/s12860-019-0195-2 (PMC6540459; doi:10.1186/s12860-019-0195-2)
Supplement: Supplementary file 2 — Table S2. Mean γ-H2AX total fluorescence yields in blood and spleen MNCs. (PDF 72 kb) [file 12860_2019_195_MOESM2_ESM.pdf]

Administered Activity (MBq)	Mean γ-H2AX total fluorescence in blood MNCs				
	2d	3d	5d	7d	14d
**0**	1018 ± 27	993 ± 39	1026 ± 35	972 ± 42	1022 ± 39
**5.74**	1356 ± 29	1288 ± 16	1215 ± 31	1157 ± 35	1087 ± 28
**6.66**	1324 ± 24	1261 ± 21	1223 ± 25	1243 ± 34	1149 ± 24
**7.65**	1257 ± 37	1258 ± 24	1084 ± 25	1120 ± 28	1106 ± 28
**9.28**	1288 ± 35	1156 ± 29	1180 ± 25	1153 ± 33	1129 ± 30
**Administered Activity (MBq)**	**Mean γ-H2AX total fluorescence in spleen MNCs**				
	2d	3d	5d	7d	14d
**0**	878 ± 16	861 ± 14	897 ± 21	905 ± 16	849 ± 22
**5.74**	972 ± 21	1073 ± 17	1045 ± 22	983 ± 18	985 ± 21
**6.66**	942 ± 12	886 ± 11	936 ± 15	1043 ± 16	979 ± 12
**7.65**	865 ± 9	843 ± 8	856 ± 12	907 ± 14	949 ± 10
**9.28**	1062 ± 14	909 ± 24	956 ± 13	881 ± 11	852 ± 8
