# Supplementary figures and images for: Effect of dose and dose rate on temporal γ-H2AX kinetics in mouse blood and spleen mononuclear cells in vivo following Cesium-137 administration
Source: BMC Mol Cell Biol. 2019 May 28;20:13. doi: 10.1186/s12860-019-0195-2 (PMC6540459; doi:10.1186/s12860-019-0195-2)

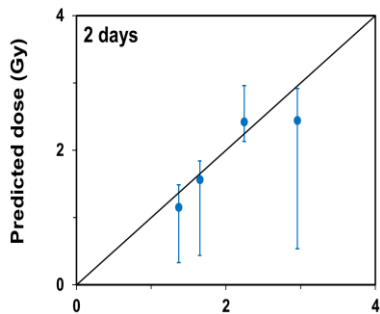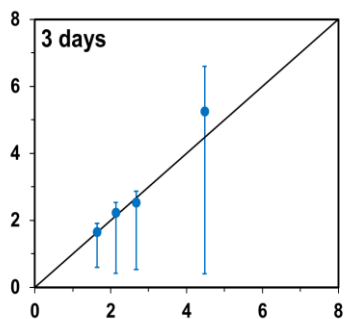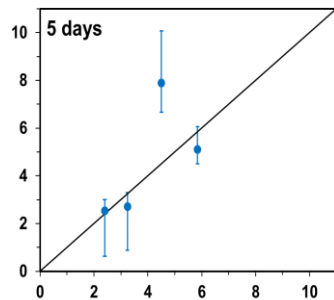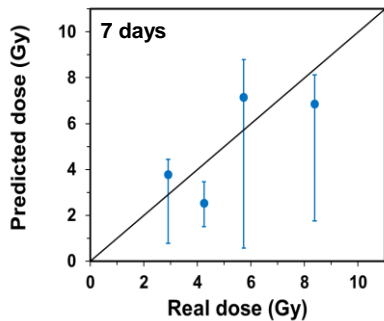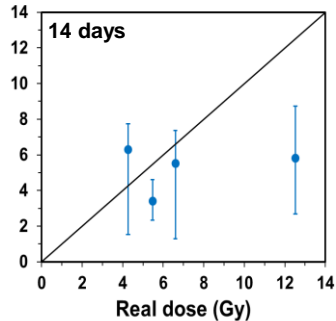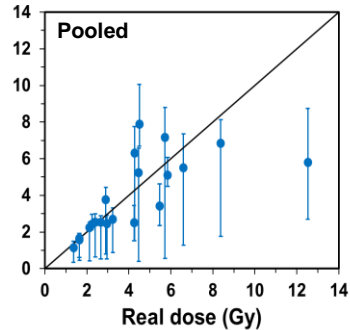

Supplement: Supplementary file 3 — Figure S1. Dose predictions calculated from injected 137Cs activity predictions at different time points. Solid circles represent median estimates across multiple Monte Carlo simulations, and error bars indicate their ranges (minimum to maximum). The black line represents a theoretical 1:1 correspondence. (PDF 140 kb) [file 12860_2019_195_MOESM3_ESM.pdf]

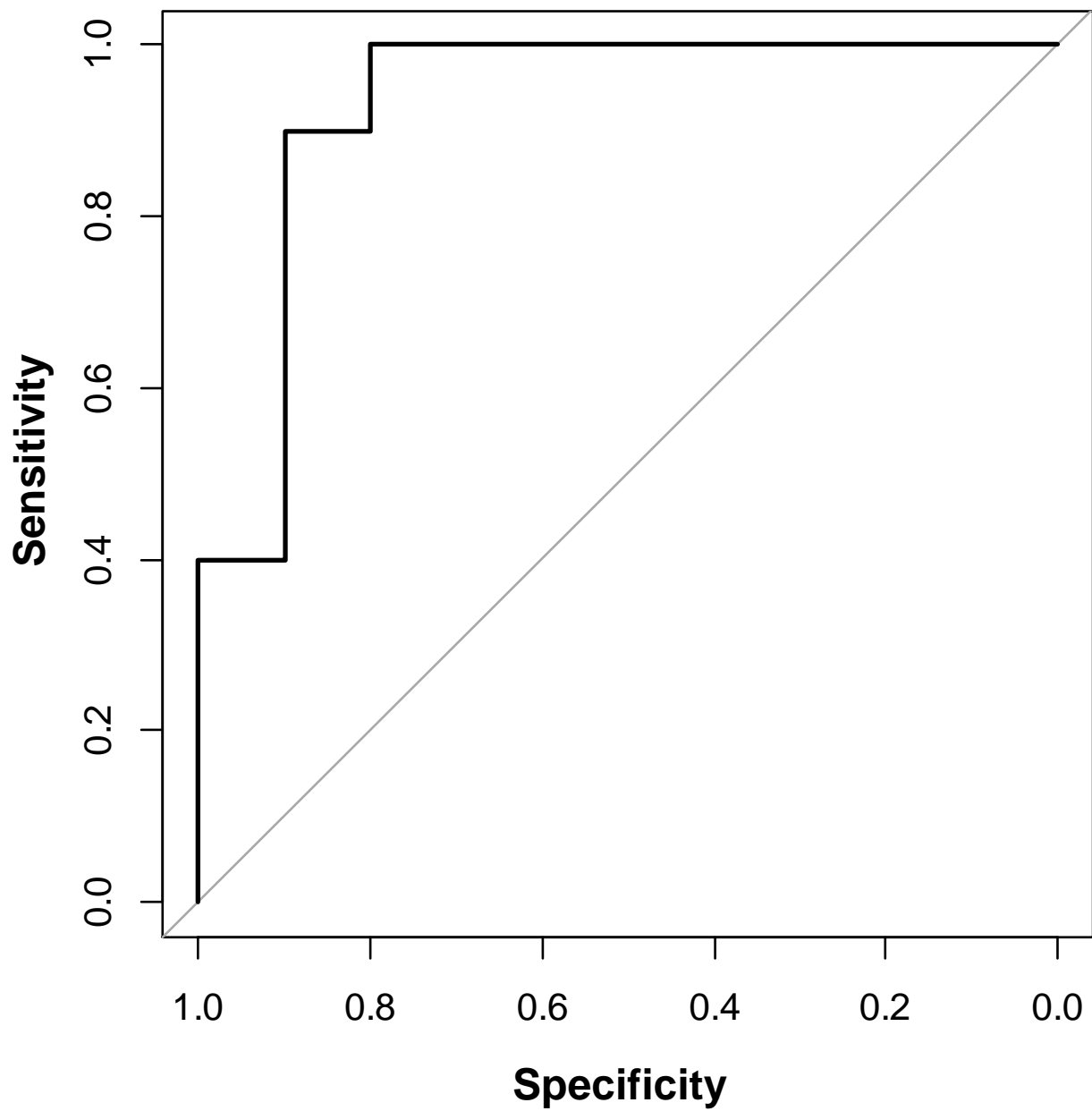

Supplement: Supplementary file 4 — Figure S2. ROC analysis of model performance on “low” vs “high” injected activities (AUC = 0.93). (PDF 4 kb) [file 12860_2019_195_MOESM4_ESM.pdf]
