# Supplementary material for: Effect of dose and dose rate on temporal γ-H2AX kinetics in mouse blood and spleen mononuclear cells in vivo following Cesium-137 administration
Source: BMC Mol Cell Biol. 2019 May 28;20:13. doi: 10.1186/s12860-019-0195-2 (PMC6540459; doi:10.1186/s12860-019-0195-2)
Supplement: Supplementary file 5 — Table S3. Measurement of the number of healthy and highly- damaged blood and spleen MNCs over the 14-day study period. For comparison, the ratio of damaged/healthy cells in non-exposed blood and spleen MNCs were 0.1; whereas the ratios of healthy/damaged cells were 17.6 and 12.3, respectively. (PDF 5 kb) [file 12860_2019_195_MOESM5_ESM.pdf]

Activity (MBq)	Blood					Spleen				
	day 2	day 3	day 5	day 7	day 14	day 2	day 3	day 5	day 7	day 14
**Highly damaged/Healthy**										
**5.74**	0.0	1.2	1.0	2.2	0.0	0.6	0.5	1.1	0.6	1.4
**6.66**	9.0	2.5	1.5	2.0	0.8	0.5	1.0	0.6	0.8	0.3
**7.65**	0.3	6.0	0.5	0.0	4.0	0.2	0.2	0.0	0.1	0.5
**9.28**	0.3	0.5	0.4	0.3	3.0	0.9	0.1	0.1	0.2	0.1
**Healthy/Highly damaged**										
**5.74**	1.0	0.9	1.0	0.5	1.0	1.6	2.0	0.9	1.8	0.7
**6.66**	0.1	0.4	0.7	0.5	1.2	2.1	1.0	1.7	1.3	3.1
**7.65**	3.3	0.2	2.2	4.0	0.3	4.9	4.3	97.0	8.8	2.1
**9.28**	4.0	2.2	2.9	4.0	0.3	1.1	14.5	9.0	4.8	9.3
